# Cell lines generated from a chronic lymphocytic leukemia mouse model exhibit constitutive Btk and Akt signaling

**DOI:** 10.18632/oncotarget.18234

**Published:** 2017-05-26

**Authors:** Simar Pal Singh, Saravanan Y. Pillai, Marjolein J.W. de Bruijn, Ralph Stadhouders, Odilia B.J. Corneth, Henk Jan van den Ham, Alice Muggen, Wilfred van IJcken, Erik Slinger, Annemieke Kuil, Marcel Spaargaren, Arnon P. Kater, Anton W. Langerak, Rudi W. Hendriks

**Affiliations:** ^1^ Department of Pulmonary Medicine, Erasmus MC, Rotterdam, The Netherlands; ^2^ Department of Immunology, Erasmus MC, Rotterdam, The Netherlands; ^3^ Post graduate school Molecular Medicine, Erasmus MC, Rotterdam, The Netherlands; ^4^ Centre for Genomic Regulation (CRG), The Barcelona Institute of Science and Technology, Barcelona Spain; ^5^ Department of Virosciences, Erasmus MC, Rotterdam, The Netherlands; ^6^ Center for Biomics, Erasmus MC, Rotterdam, The Netherlands; ^7^ Department of Hematology, Academic Medical Center, Amsterdam, The Netherlands; ^8^ Department of Pathology, Academic Medical Center, Amsterdam, The Netherlands

**Keywords:** B-cell receptor (BCR), bruton’s tyrosine kinase (Btk), chronic lymphocytic leukemia (CLL), ibrutinib, idelalisib

## Abstract

Chronic lymphocytic leukemia (CLL) is characterized by the accumulation of mature CD5^+^ B cells in blood. Spontaneous apoptosis of CLL cells *in vitro* has hampered in-depth investigation of CLL pathogenesis. Here we describe the generation of three monoclonal mouse cell lines, EMC2, EMC4 and EMC6, from the *IgH.TEμ* CLL mouse model based on sporadic expression of SV40 large T antigen. The cell lines exhibit a stable CD5^+^CD43^+^IgM^+^CD19^+^ CLL phenotype in culture and can be adoptively transferred into *Rag1*^−/−^ mice. RNA-seq analysis revealed only minor differences between the cell lines and their primary tumors and suggested that NF-κB and mTOR signaling pathways were involved in cell line outgrowth. *In vitro* survival and proliferation was dependent on constitutive phosphorylation of Bruton's tyrosine kinase (Btk) at Y551/Y223, and Akt(S473). Treatment of the cell lines with small molecule inhibitors specific for Btk (ibrutinib) or PI3K (idelalisib), which is upstream of Akt, resulted in reduced viability, proliferation and fibronectin-dependent cell adhesion. Treatment of cell line-engrafted *Rag1*^−/−^ mice with ibrutinib was associated with transient lymphocytosis, reduced splenomegaly and increased overall survival. Thus, by generating stable cell lines we established a novel platform for *in vitro* and *in vivo* investigation of CLL signal transduction and treatment modalities.

## INTRODUCTION

Chronic lymphocytic leukemia (CLL) is characterized by the accumulation of monoclonal mature B cells with a CD5^+^CD19^+^CD20^dim^Ig^dim^CD23^+^CD43^+^CD27^+^ surface phenotype in the circulation [1, 2]. Several lines of evidence support a key role for B cell receptor (BCR) signaling in CLL pathogenesis. First, CLL with hypermutated immunoglobulin heavy chain variable (IGHV) genes (M-CLL) show a more favorable prognosis than those with unmutated IGHV genes (U-CLL) [3, 4]. Secondly, the IGHV repertoire is highly restricted, whereby stereotypic BCRs are found in multiple CLL patients [5]. Thirdly, CLL B cells often show increased basal activity of protein tyrosine kinases downstream of the BCR [6, 7]. Hereby, Bruton's tyrosine kinase (Btk) has been shown to be essential for several constitutively active pathways implicated in CLL cell survival, including the Akt, ERK and NF-κB pathway [8–11]. Antitumor activity of the Btk small-molecule inhibitors ibrutinib and acalabrutinib was recently shown in clinical studies of relapsed/refractory CLL [12, 13]. Furthermore, CLL B cells manifest low surface IgM (sIgM) expression and their BCR signaling properties resemble those of anergic B cells. During *in vitro* culture they readily upregulate sIgM expression and regain BCR responsiveness [14–17]. Accordingly, CLL B cells have higher basal, cell-autonomous Ca^2+^ signaling, dependent on an internal BCR epitope [18, 19]. Alternatively, the recent identification of antigen-specificity of particular CLL B cells indicates that their proliferation and survival is driven by specific (auto)antigens [20, 21].

CLL B cells are thought to interact with the tissue microenvironment [22–24] and lymph node resident CLL cells show gene expression signatures indicative of BCR activation [25]. Btk may be involved in trafficking of CLL B cells to survival niches, because it also functions downstream of chemokine receptors such as CXCR4 and CXCR5 [11] and has been implicated in *in vivo* homing to lymphoid organs [26]. Accordingly, treatment of CLL cells with ibrutinib inhibited CXCL12/CXCL13-induced *in vitro* cell adhesion and migration [27, 28] and in CLL patients ibrutinib treatment resulted in a transient lymphocytosis, further underscoring the role of Btk in CLL-cell trafficking and homing [12].

Given the importance of intrinsic BCR signaling for survival and progression of CLL as well as support from the tumor microenvironment, research into CLL pathogenesis would benefit from systems that can explore both pathways. However, these approaches have been hampered by the limited *in vitro* survival and non-dividing characteristics of human CLL B cells. Those few available cell lines derived from CLL patients (CD5^−^ MEC1 and MEC2 [29], PCL12 [30], OSU-CLL [31] and MDA-BM5 [32]) may represent EBV^+^ B-lymphoblastoid cells rather than bonafide B-CLL cells.

Mouse models have provided important insights into CLL pathogenesis. These particularly include the widely studied Eμ-TCL1 model, in which B-cell specific overexpression of the *TCL1* oncogene results in spontaneous development of leukemic CD5^+^IgM^+^ B cells [33–35]. Effects of ibrutinib or the Syk inhibitor fostamatinib (R406) on Eμ-TCL1 leukemias have been tested, whereby the outcome mimicked clinical observations in patients [28, 36]. Another mouse model (*IgH.TEμ*) was generated in our lab and is based on sporadic expression of the SV40 large T oncogene in mature B cells [37]. This was achieved by SV40T insertion in opposite transcriptional orientation into the *IgH* locus D_H_-J_H_ region. Aging *IgH.TEμ* mice show accumulation of monoclonal leukemic CD5^+^CD43^+^IgM^+^IgD^low^CD19^+^ B cells, which is dependent on Btk expression and whereby Btk-mediated signaling enhances leukemogenesis [37, 38].

Despite their proven usefulness as pre-clinical tools, transgenic mouse models take substantial time (> 6 months) to develop CLL and are not suitable for large-scale screens of novel compounds or combination therapies. Therefore, we aimed to obtain stable CLL cell lines that can be cultured *in vitro* or transferred into mice *in vivo.* In addition, we aimed to explore whether these cell lines could serve as a platform for the investigation of CLL signal transduction and to investigate the efficacy of small molecule inhibitor combinations in CLL. Here, we describe the generation and characterization of three monoclonal CD5^+^CD43^+^IgM^+^CD19^+^ cell lines from *IgH.TE*μ mice. Parallel to human CLL, our cell lines exhibited constitutive activation of BCR downstream kinases.

## RESULTS

### Generation and characterization of cell lines from IgH.TEμ CLL mice

To obtain stable cell lines, single cell suspensions from spleens of aged *IgH.TE*μ mice [37] with high tumor load (> 90% CD5^+^CD43^+^IgM^+^CD19^+^ cells) were cultured under various conditions, with or without BAFF, a-CD40 antibodies and rIL-4. After 8 weeks outgrowth was observed in three independent cultures: EMC2, EMC4 and EMC6. The presence of BAFF, a-CD40 or rIL-4 did not appear to be critical and after the initial few passages the EMC cell lines were expanded in culture medium without supplements.

**Figure 1:**
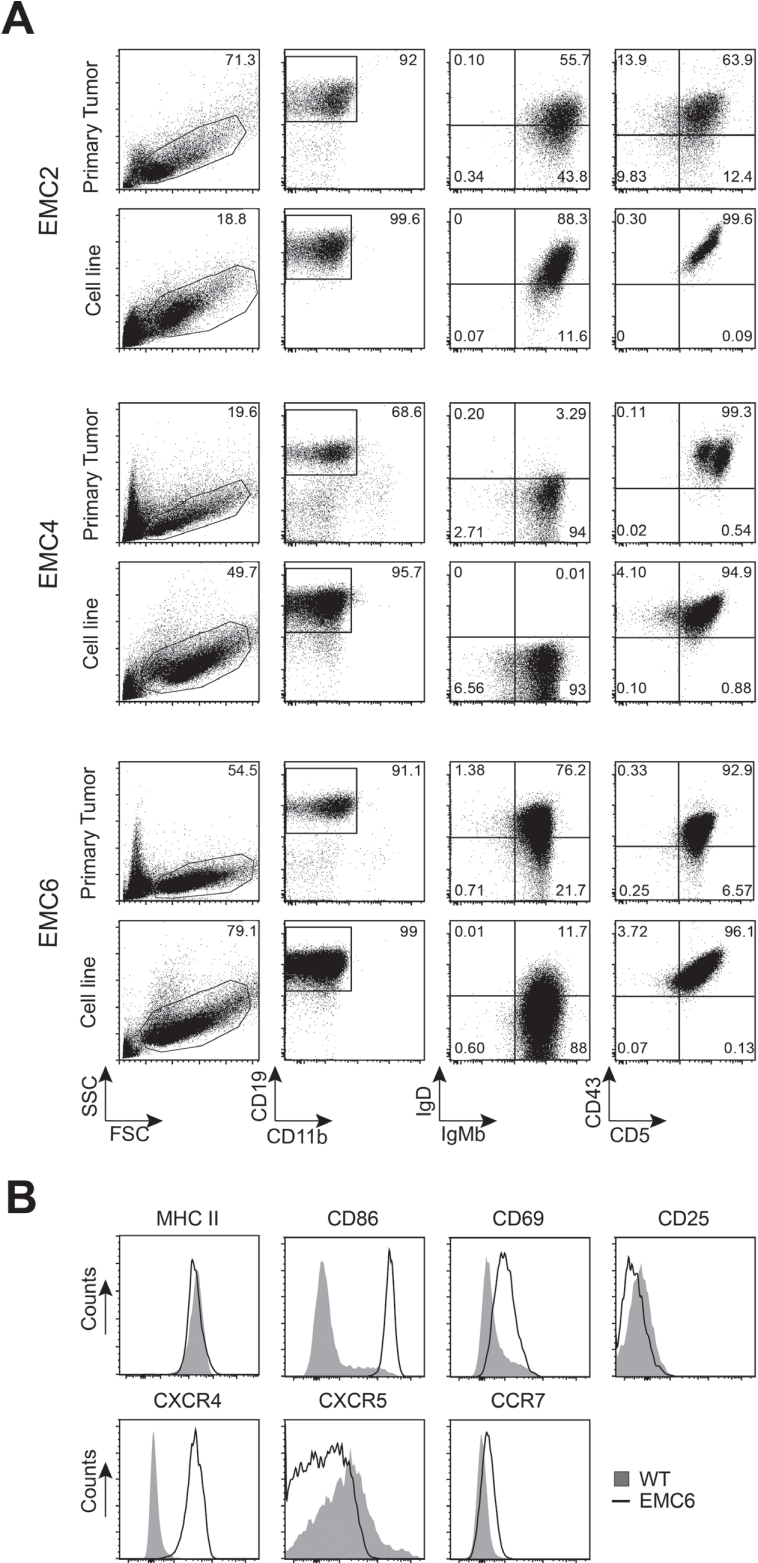
EMC cell lines resemble primary tumors from *IgH.TEμ* mice (**A**) Phenotypic comparison of CLL cells from primary splenic tumor cells and established cell lines by flow cytometry. Gated CD11b^−^CD19^+^(*second column*) were analyzed for IgM/IgD (*third column*) and CD5/CD43 (*fourth column*). (**B**) Histograms showing expression of the indicated markers on gated CD19^+^ WT splenic cells (*n* = 4) and EMC6 cells, determined by flow cytometry. EMC4 and EMC2 showed similar expression profiles, unless indicated in text.

Flow cytometry analysis showed that the EMC cell lines maintained the CD5^+^CD43^+^IgM^+^CD19^+^ phenotype of the primary leukemia [37], even after prolonged (at least 22 weeks) *in vitro* culture (Figure 1A, Supplementary Figure 1A). Expression levels of the activation markers CD69 and CD86 were higher on the EMC cell lines than on control wild type (WT) splenic B-cells, but surface MHCII or CD25 was similar (shown for EMC6 in Figure 1B, Supplementary Figure 1A). Compared with WT B cells, the EMC cell lines exhibited stronger expression of CXCR4 and CCR7, but not CXCR5 (Figure 1B; Supplementary Figure 1A, 1B). The expression profiles of activation markers and chemokine receptors of the EMC cell lines resembled those of primary tumors from *IgH.TEμ* mice (*n* = 20), except for CD69 when compared to WT B cells (Figure 1B ; Supplementary Figure 1C).

Thus, we generated three stable cell lines that maintained the CD5^+^CD43^+^IgM^+^CD19^+^ phenotype of the primary CLL, even after prolonged *in vitro* culture.

### RNA sequencing reveals limited differences between EMC cell lines and their corresponding primary leukemias

**Figure 2:**
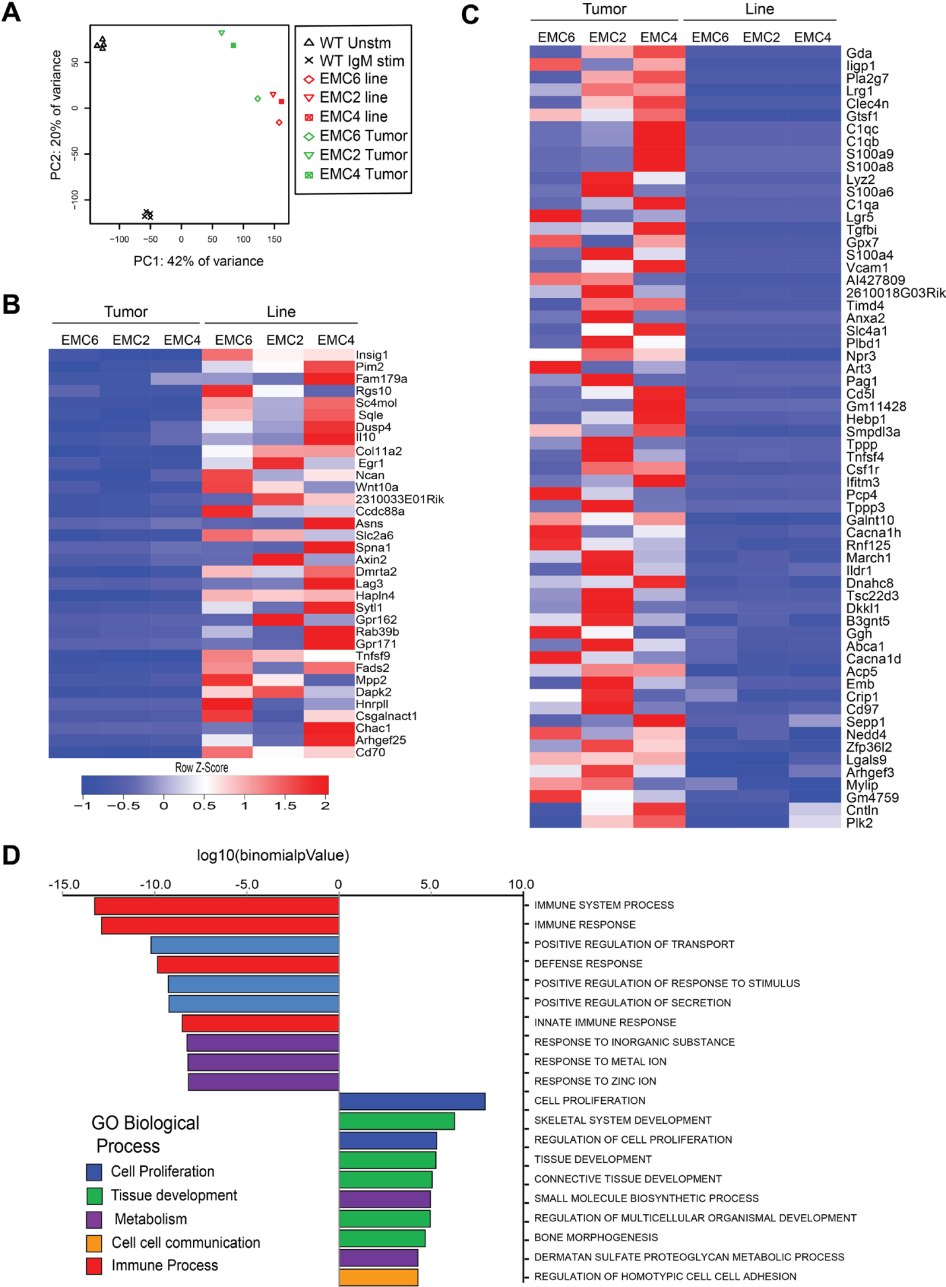
RNA-sequencing reveals limited differences between EMC cell lines and primary leukemias (**A**) Principle component analysis comparing genome-wide RNA-Seq profiles in resting (Unstim, *n* = 4) or a-IgM-stimulated (IgM stim, *n* = 4) WT splenic B cells, primary tumors (green, *n* = 3) and EMC cell lines (red, *n* = 3). (**B**, **C**) Heat map showing variation in levels (Z-Score) of 34 upregulated (B) and 62 downregulated genes (C) in the EMC lines compared to primary tumors, ordered by *p*-value (Supplementary Table 1) (**D**) Top 20 biological processes enriched among differentially expressed genes in EMC cells and primary tumors (from MSigDB database).

To identify pathways involved in the outgrowth of the three cell lines from the corresponding primary tumors, we compared genome-wide RNA-seq gene expression profiles. We included resting and a-IgM-stimulated WT splenic B cells as controls. A principle component analysis (Figure 2A) revealed substantial differences between resting and activated WT splenic B cells and primary CLL. The three EMC cell lines clustered together, close to the primary tumors, with EMC6 showing the smallest difference to its corresponding primary leukemia (Figure 2A).

Next, we performed differential gene expression analysis to identify 246 genes that showed more than 2-fold change between primary tumors and EMC cell lines, and from these we subsequently selected genes with FPKMs >1 in at least 2 samples. We identified 34 upregulated and 62 downregulated genes in the EMC cell lines, compared with the primary tumors (Figure 2B, 2C; See Supplementary Table 1A, 1B for expression values). Expression levels were confirmed by QPCR for a number of key genes, including Tnfsf9, Pim2, CD70 and Egr1, which are also highly expressed in human CLL [39–43] (Supplementary Figure 2A, 2B). Interestingly, the glucocorticoid-induced leucine zipper protein (GILZ), encoded by the *Tsc22d3* gene, which inhibits the mTORC/AKT signaling pathway [44] was amongst the genes downregulated in the EMC cell lines (Supplementary Figure 2C). We did not find upregulation of anti-apoptotic genes, including Bcl-2, Bcl-XL or Mcl-1 in the EMC cell lines, compared to primary leukemias. However, expression of these anti-apoptotic genes was increased in both primary tumors and EMC cell lines, when compared to control WT splenic B cells (Supplementary Figure 2D).

The top 20 biological processes (Molecular Signatures Database MsigDB) [45] enriched within the differentially expressed genes in the EMC cell lines showed an overrepresentation of cell proliferation, metabolic and tissue development-related pathways (Figure 2D). Moreover, pathway analysis revealed a significant overlap with a gene set upregulated in “CLL expressing naturally phosphorylated CD5” (Insig1, Sqle, Tnfsf9, Asns, Pim2, Wnt-10A, IL-10, CD70, Rab39b) [46]. Other prominent signatures emerging from genes upregulated in the EMC cell lines compared to primary CLL tumors were “mTorc1 signaling” and “TNFα signaling via NFkB” (both with *p* = ∼10^−5^, FDR<0.05; Supplementary Figure 2C).

In conclusion, the genome-wide expression profiles of the three cell lines closely resembled those of the primary tumors and suggest that mTorc1 signaling contributed to cell line outgrowth.

### EMC cell lines express a VH11 BCR recognizing PtC and exhibit constitutive BCR signaling

**Figure 3:**
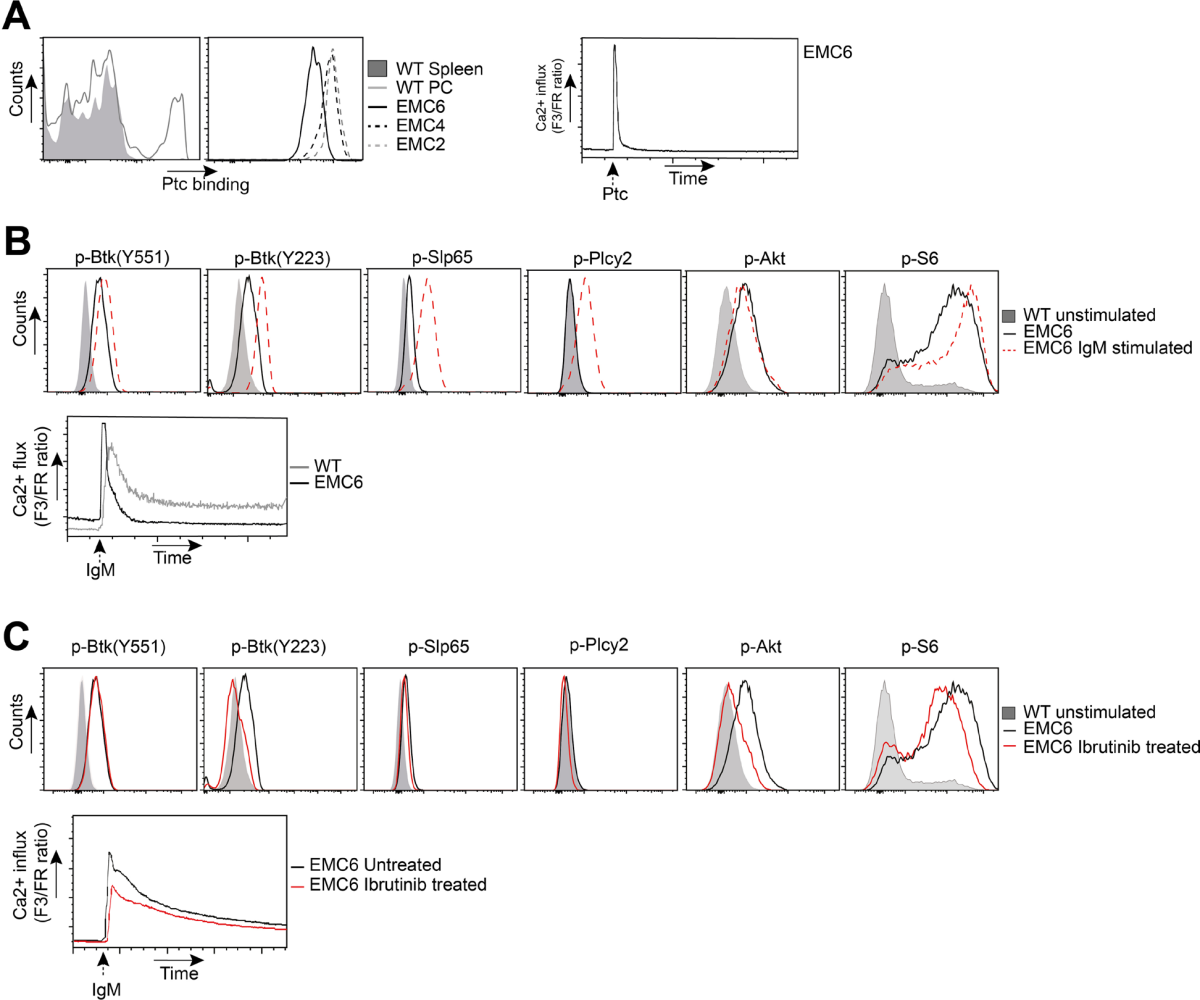
EMC cell lines recognize autoantigens and exhibit constitutive BCR signaling (**A**) *Left*: Flow cytometric analysis of gated wildtype (WT) CD19^+^ splenocytes and CD19^+^CD5^+^ peritoneal cavity (PC) cells, and EMC cell lines stained with phosphatidylcholine (Ptc) liposomes. *Right*: Ca^2+^-flux analysis of PtC-stimulated EMC6. (**B**) *Top*: PhosFlow analysis of indicated phosphoproteins on gated unstimulated B220^+^CD3^−^ WT splenocytes and EMC6 cells, and a-IgM-stimulated (20 μg/ml) EMC6 cells. *Bottom*: Comparison of basal or a-IgM stimulated Ca^2+^ influx between B220^+^CD3^−^ WT splenic B cells and EMC6. (**C**) *Top*: PhosFlow analysis of indicated phosphoproteins on gated unstimulated B220^+^CD3^−^ WT splenocytes and EMC6 cells and 1 mM ibrutinib-treated EMC6 cells. *Bottom*: Comparison of Ca^2+^-influx between untreated or 1 mM ibrutinib-treated EMC6 cells. For all analyses EMC6 cells are shown as representative of the three cell lines.

Next, we determined the rearranged IGHV and IGLV DNA sequence of the EMC cell lines. Even though generated independently from three different mice, all three EMC cell lines expressed V_H_11.2*01 (HCDR3: CMRYSNYWYFDVW) together with V_K_14-126*01 (LCDR3: CLQHGESPYTF), which was identical to the corresponding primary tumors. This stereotypic V_H_11/V_K_14 BCR is known to be specific for phosphatidylcholine (PtC) [47] and is the prominent BCR found in both Eμ-TCL1 transgenic CLL [21] and in *IgH.TEμ* transgenic CLL [37, 38]. We could confirm uniform PtC binding of EMC cell lines, yielding similar staining intensities as WT peritoneal cavity B-1 cells (Figure 3A). Hereby, the signal for EMC6 was lower than for EMC2 and EMC4. PtC also induced Ca^2+^ flux in all three EMC cell lines, indicating that their BCRs did not only bind but also responded to PtC (Figure 3A).

Next, we compared the activation status of BCR downstream signaling proteins between the cell lines and WT splenic B cells by intracellular flow cytometry for phosphorylated proteins (Phosflow). In the absence of external stimulation, the EMC cell lines exhibited significant phosphorylation of Btk (Y551;Y223), Slp65(Y84), Akt(S473) and its target S6 (Figure 3B, Supplementary Figure 3D). As phosphorylation of these proteins was not observed in resting B cells, these findings provided evidence for constitutively active BCR/Akt signaling in the EMC cell lines, which was corroborated by increased basal Ca^2+^ signals (Figure 3B, Supplementary Table 3). Importantly, the constitutive BCR/Akt signaling in the EMC cell lines was stable over 22 weeks of *in vitro* culture (Supplementary Figure 3E). In line with our previous report [37, 38], constitutively active Btk signaling was not apparent in primary *IgH.TEμ* CLL cells, but these cells had low but detectable expression of p-Akt and substantial levels of p-S6 (Supplementary Figure 3A). Anti-IgM stimulation of EMC cell lines further increased p-Btk(Y551/Y223), p-Slp65(Y84), induced p-Plcγ2(Y759), but did not further increase p-Akt(S473) expression (Figure 3B). Thus, in the EMC cell lines constitutive Btk activation was suboptimal and did not result in detectable Plcγ2(Y759) phosphorylation, but could be further enhanced by BCR stimulation. In addition, we found that the EMC cell lines had high basal Ca^2+^ and less sustained Ca^2+^ elevation in response to a-IgM stimulation, compared with WT B cells (Figure 3B), which represent key features of the anergic phenotype of human CLL B cells [14–17]. Because of the similarities between the EMC cell lines, for most of the experiments described below, we focused on EMC4 and EMC6.

Next, we investigated whether constitutively active BCR signaling in the EMC cell lines was dependent on Btk kinase activity. We found that in the presence of ibrutinib the signals specific for p-Btk(Y223) but not for p-Btk(Y551) were substantially reduced (Figure 3C). Whereas the effect of ibrutinib treatment on lowering basal p-Slp65 was less apparent, it clearly downregulated p-Akt(S473) and pS6. In the presence of ibrutinib, the a-IgM induced Ca^2+^ influx was also slightly reduced (Figure 3C, Supplementary Figure 3B). Constitutive Btk(Y551) phosphorylation was Syk kinase independent, because addition of the Syk inhibitor R406 did not affect p-Btk(Y551), nor p-Akt(S473) signals (Supplementary Figure 3C). However, R406 did decrease p-Slp65(Y84), p-Plcγ2(Y759) and a-IgM induced Ca^2+^ influx (Supplementary Figure 3C).

Collectively, these findings show that the EMC cell lines are monoclonal, express a PtC-specific V_H_11/V_K_14 BCR and exhibit stable constitutive Btk and Akt activation. Constitutive Btk(Y551) phosphorylation was Syk-independent and Btk(Y223) autophosphorylation was suboptimal. Whereas upon a-IgM stimulation of the EMC cell lines p-Slp65(Y84) and p-Plcγ2(Y759) were induced, Ca^2+^ influx was limited, congruent with an anergic response to BCR stimulation.

### EMC cell lines provide a novel *in vitro* tool to test therapeutic drugs for CLL

The prolonged stability of the EMC cell lines in culture enabled us to test their sensitivity towards different classes of DNA damaging agents. The cell lines were relatively resistant to Fludarabine (LC50 = 17 μM), a purine analogue widely used in combination regimens in CLL. The cells turned out to be highly resistant to cisplatin (LC50 = 9.73 μM), which is used in combination regimens in chemorefractory patients. These data are compatible with p53 dysfunction in *IgH.TE*μ mice [37] and lack of induction of the p53 target gene Puma following 24 h of treatment [48]. Interestingly, the EMC cell lines were sensitive to the topoisomerase II inhibitor etoposide (LC50 = 0.24 μM) (Supplementary Figure 4A, 4B), allowing *in vitro* testing of combination strategies including genotoxic agents.

**Figure 4:**
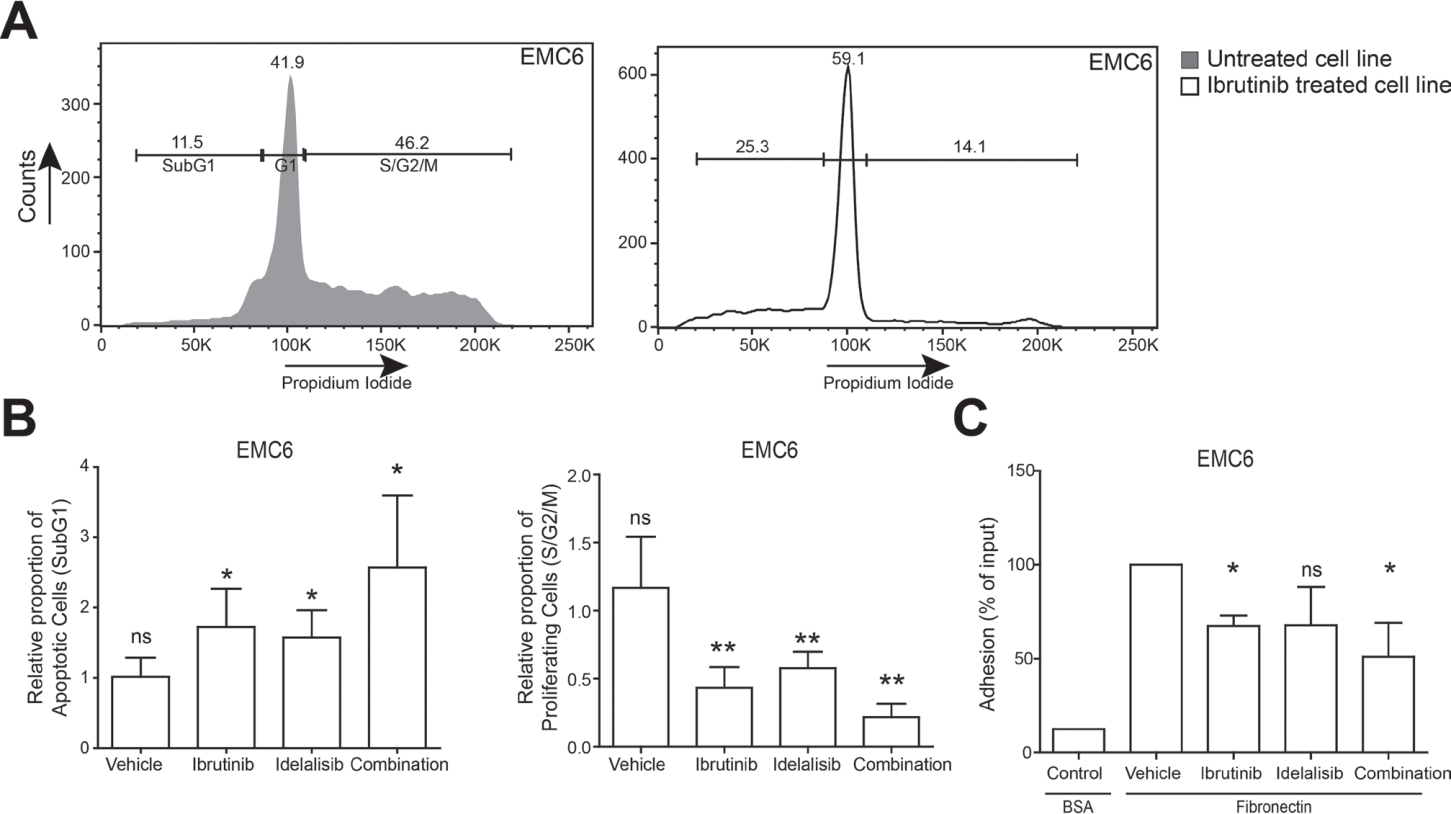
EMC cell lines provide novel *in vitro* tools to test therapeutic drugs for CLL (**A**) Gating strategy for DNA content (Propidium Iodide) analysis of EMC6 cells after 24 h culture in the absence or presence of Ibrutinib. Numbers indicate proportions of cells in the respective gates. (**B**) Relative proportions of cycling (S/G2-M) and apoptotic (sub-G1) EMC6 cultured (*n* = 3, in duplicate) in the presence of either ibrutinib (5 nM), idelalisib (100 nM) or in combination. Graphs are presented as normalized mean ± SEM (Untreated EMC6 cells were set to 1). (**C**) *In vitro* adhesion to fibronectin. EMC6 cells were pretreated with ibrutinib (10 nM), idelalisib (100 nM) or a combination (*n* = 3, in triplicate). Graphs are presented as normalized mean ±SD (100%= vehicle-treated EMC6 cells). *P < 0.05, **P < 0.01 (paired one-sample *T*-test).

To investigate the role of constitutively active Btk and Akt on EMC cell viability and proliferation, we performed propidium iodide DNA content analyses following overnight treatment with ibrutinib, idelalisib or vehicle as controls. In the presence of ibrutinib and idelalisib the proportions of apoptotic (sub-G1) and cycling (S/G2/M) cells were significantly increased and decreased, respectively (Figure 4A, 4B). To quantify the effect of combination treatment, we calculated combination index (CI) values [49] and observed synergistic effects (CI value < 1) on viability and proliferation when the two inhibitors were combined. Interestingly, ibrutinib induced a G1 cell-cycle arrest in EMC6 (Figure 4A). Therefore, we evaluated the impact of ibrutinib on different families of apoptosis and proliferation regulators. Ibrutinib treatment alone showed >2 fold downregulation of survivin mRNA expression (Supplementary Figure 4C–4F), which was highly expressed in both primary tumors and EMC cell lines, when compared to control WT splenic B cells (Supplementary Figure 2D). Since survivin has been shown to be downstream of the PI3K/Akt pathway in CLL [50], it further validates the importance of constitutive Akt signaling in the EMC cell lines. Ibrutinib or idelalisib also significantly inhibited integrin-mediated adhesion to fibronectin *in vitro,* again showing a synergistic effect (CI value < 1) when these inhibitors were combined (Figure 4C, Supplementary Figure 4G).

Collectively, these results show the importance of constitutively active Btk and Akt signaling for survival, proliferation and adhesion of the EMC cell lines in *in vitro* cultures.

### Engraftment of EMC cell lines into Rag1^−/−^ mice as a tool to study novel CLL therapeutics

**Figure 5:**
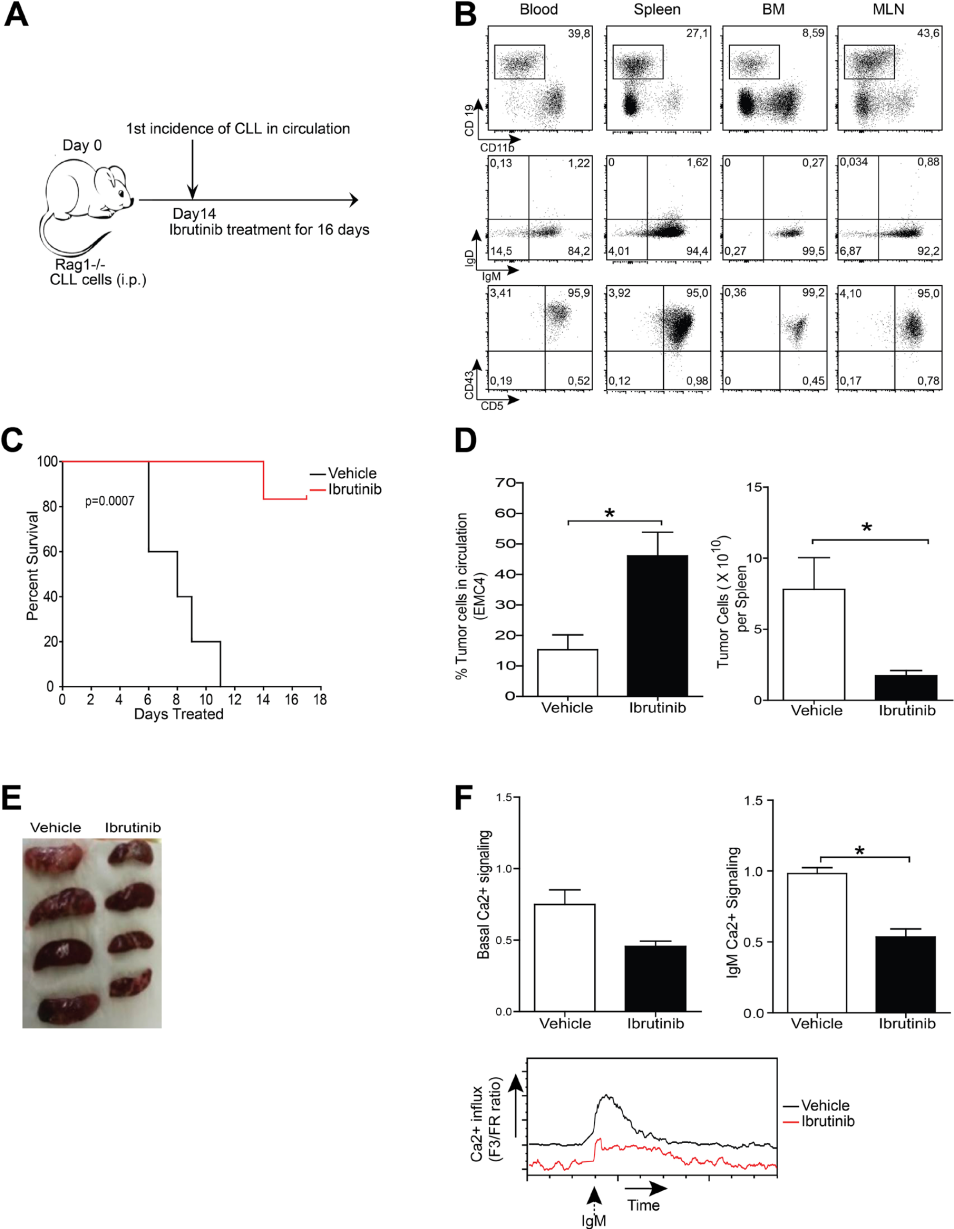
Leukemia induction following EMC cell engraftment as a tool to study therapeutics (**A**) Experimental timeline of EMC cell transfer. (**B**) Flow cytometric analysis of blood, spleen, bone marrow (BM) and mesenteric lymph node (MLN) of *Rag1^−/−^* mice after 4 weeks of engraftment of 5×10^6^ EMC4 cells. CD19+CD11b- cells were gated (*left column*) and analyzed for IgM/IgD and CD5/CD43 in the indicated tissues. Dot plots are representative of successful EMC cell engraftment. (**C**) Kaplan-Meier survival curve over 16 days of ibrutinib or vehicle treatment of EMC6- engrafted *Rag1^−/−^* mice (*n* = 8 mice/group). (**D**) Proportions of circulating leukemic cells (*left*) and absolute number of leukemic cells in spleen (*right*), 16 days after treatment of EMC4-engrafted mice with vehicle or ibrutinib (*n* = 4 mice/group). (**E**) Spleens from EMC4-engrafted mice treated for 16 days with vehicle or ibrutinib. (**F**) Comparison of basal and a-IgM-stimulated (20 μg/ml) Ca^2+^-influx *ex vivo* on splenic tumor cells from EMC4-engrafted mice treated for 16 days with vehicle or ibrutinib. Representative (*n* = 4) MFI kinetics plot of Ca^2+^ signaling (*bottom*) from each group.*P < 0.05 (*n* = 4 mice/group, Mann-Whitney *U* test).

To test their tumorigenic potential, we transferred 1–5 × 10^6^ EMC cells into *Rag1^−/−^* mice [51] by i.p. injection (Figure 5A). From 2 weeks post-engraftment onwards, a population of CD5^+^CD43^+^IgM^+^CD19^+^ B cells was detectable; these cells were not only present in peripheral blood, but also in various lymphoid organs, including spleen, bone marrow and mesenteric lymph node (shown for EMC4-engrafted mice, 4 weeks after transfer in Figure 5B).

To test the effects of ibrutinib *in vivo,* we compared mouse survival in EMC6-engrafted mice that received either ibrutinib or vehicle. Two weeks after engraftment mice were randomized and ibrutinib or vehicle treatment was initiated and continued for 16 days (Figure 5A). Whereas in the vehicle group mice had to be euthanized as their condition went down rapidly, a major proportion (∼80%) of the ibrutinib treatment group was still alive after 16 days of treatment (Figure 5C).

Next, we investigated if ibrutinib treatment affected disease progression. EMC4-engrafted mice were randomized to receive either ibrutinib or vehicle. After 3 and 12 days of treatment, the proportions of circulating CD5^+^CD43^+^IgM^+^CD19^+^ B-cells were not different between the two groups (Supplementary Figure 5). However, at the end of the 16-day treatment cycle, a major proportion of the control group exhibited lethargy, hunched posture or other disabling symptoms. Therefore, animals from both arms were sacrificed to compare disease progression. The ibrutinib-treated group had a significant increase of circulating tumor cells, compared with the vehicle-treated group (Figure 5D). Vehicle-treated mice had massive splenomegaly, while ibrutinib-treated mice had smaller spleens with significantly lower leukemic CD5^+^CD43^+^IgM^+^CD19^+^ B cell counts (Figures 5D and 5E). No significant differences were found in the leukemic cell counts in bone marrow and mesenteric lymph nodes in the two groups (data not shown). Interestingly, ibrutinib-treated mice displayed lower basal Ca^2+^ signaling and significantly reduced Ca^2+^ influx following a-IgM stimulation, compared with vehicle-treated control mice (Figure 5F).

Thus, our data demonstrate that we have established a novel *in vivo* CLL engraftment model by adoptive transfer of EMC cell lines into *Rag1^−/−^* mice. Importantly, EMC cells remained sensitive to ibrutinib treatment *in vivo*.

### EMC4 Leukemic cells acquire an anergic phenotype when engrafted

Leukemic B cells from the spleens of EMC4-engrafted mice showed only limited induction of Ca^2+^ flux upon a-IgM stimulation *ex vivo* (Figure 5F), which was in stark contrast to our previous observation of a substantial a-IgM driven Ca^2+^ flux in cultured EMC4 cells (Supplementary Figure 3B).

**Figure 6:**
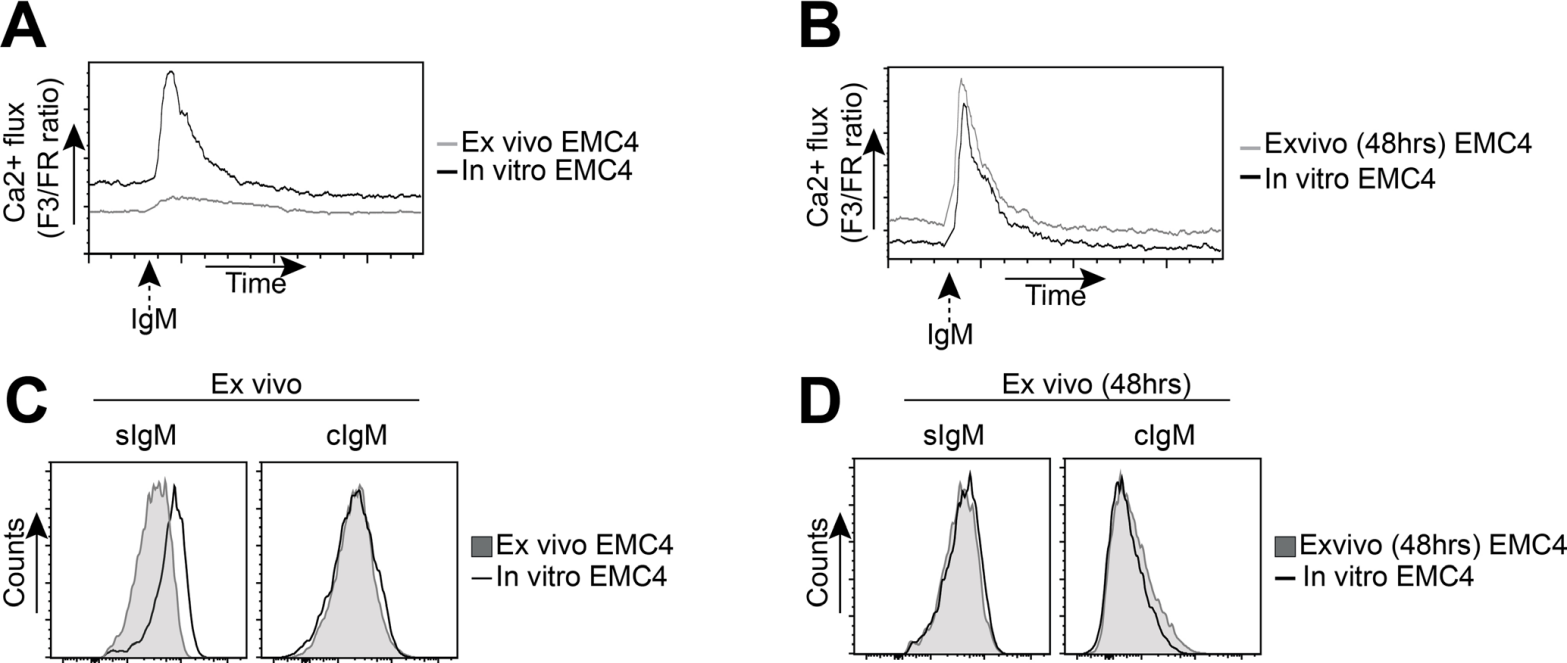
EMC4 cell lines acquire a more anergic phenotype upon engraftment (**A**, **B**) Flow cytometry analysis of a-IgM-stimulated (20 μg/ml) Ca^2+^-influx between leukemic cells, directly isolated from EMC4-engrafted mice (A, *grey line*) or after 48 hours of culture (B, *grey line*), compared with *in vitro* cultured (non-transferred) EMC4 cells (A,B: *solid black line*). (**C**, **D**) Expression of surface IgM (sIgM) and cytoplasmic IgM (cIgM) between CD5^+^CD19^+^CD43^+^ splenic leukemic cells from EMC4-engrafted mice analyzed directly after isolation (C, *shaded area*) or after 48 hours of culture (D, *shaded area*), compared with an *in vitro* cultured (non-transferred) EMC4 cells (*solid black line*).

Therefore, we compared a-IgM-induced Ca^2+^ flux between leukemic cells isolated directly from spleens of EMC4-engrafted mice (*ex vivo*) with long-term *in vitro* cultured EMC4 cells. Unlike *in vitro* cultured EMC4 cells, the *ex vivo* EMC4 cells from engrafted mice showed limited induction of intracellular Ca^2+^ flux upon BCR stimulation (Figure 6A). However, when these EMC4 cells isolated from engrafted mice were cultured for 48h, they regained their BCR responsiveness and Ca^2+^ flux was similar to *in vitro* cultured EMC4 cells (Figure 6B).

Interestingly, the freshly isolated leukemic cells from EMC4-engrafted mice showed low sIgM expression compared with *in vitro* cultured EMC4 cells (Figure 6C, *left*). Upon 48h culturing of the isolated leukemic cells, this difference in sIgM expression with *in vitro* cultured EMC4 cells was no longer detected (Figure 6D, *Left*). The sIgM upregulation was already apparent at 24h (data not shown). We did not see any difference in the cytoplasmic IgM (cIgM) expression (Figure 6C, 6D, *right*). We therefore conclude that EMC4 cells become anergic to BCR stimulation following engraftment in *Rag1^−/−^* mice, whereby they downregulate sIgM expression.

## DISCUSSION

Proliferation and survival of CLL B-cells are thought to be regulated by intracellular signaling pathways activated by various stimuli from the microenvironment. However, spontaneous apoptosis of CLL cells when cultured *in vitro* has hampered CLL research. Here, we describe the generation of three monoclonal mouse cell lines (EMC2, EMC4 and EMC6) from the SV40 large T antigen-based *IgH.TEμ* CLL mouse model. These cell lines can be cultured *in vitro* for long periods of time and transferred into mice, thus providing a platform to study BCR signaling in CLL and to investigate the efficacy of small molecule inhibitors. The EMC cell lines were remarkably similar to the primary tumors and our analyses indicate that the major pathways associated with their *in vitro* outgrowth are downstream of the BCR (Btk and Akt), CD5 and TNFa/NFkB. Stereotypic BCRs with specific IGHV usage [5] and reactivity towards common antigens [20, 21] are characteristic features of CLL. It was remarkable that all three EMC cell lines, even though generated from three different mice, express the stereotypic Ig VH11/Vκ14 genes and recognize PtC. Moreover, we did not see outgrowth of other non-VH11 clones *in vitro*. This may suggest that self-reactivity might drive the selection and outgrowth of these cell lines in *in vitro* cultures. Recently, the usefulness of the stable human CLL cell lines MEC-1 & MEC-2 to test effects of kinase inhibitors such as ibrutinib was shown [52]. Nevertheless, these cell lines are derived from EBV^+^ B cells and do not stably express surface CD5 and CD43 [29]. In contrast, EMC cell lines show stable surface expression of CD5 and intact downstream signaling, as several known downstream CD5 targets were upregulated.

Human CLL cells show aberrant BCR signaling, whereby the downstream Btk/Plcγ2/Ca^2+^ and PI3K/Akt pathways are thought to be constitutively activated, resulting in increased proliferation and survival [8–10]. We show that our EMC cell lines exhibit *in vitro* - thus in the absence of any stimulatory signals from the microenvironment - constitutive activation of p-Btk(Y551/Y223) and downstream basal Ca^2+^ signaling, paralleling human CLL. However, constitutive Btk signaling was apparently suboptimal, given the lack of phosphorylation of its main substrate PLCγ2(Y759), and was associated with BCR hyporesponsiveness. BCR anergy was also evident *in vivo*, because EMC4-engrafted cells downregulated sIgM and lacked a-IgM-induced Ca^2+^ influx. Additionally, EMC cells exhibited constitutively active Akt/S6 signaling, possibly because of the ability of SV40T protein to induce cell survival via Akt [53]. This is remarkably similar to the most extensively studied CLL mouse model, Eμ-TCL1, in which TCL-1 expression has also been functionally linked to enhanced Akt signaling [34]. However, to the best of our knowledge stable Eμ-TCL1 leukemia-derived cell lines have not been reported to date.

Evidence is emerging that the efficacy of the novel CLL therapeutics ibrutinib and idelalisib is not only based on their effects on BCR-mediated survival and proliferation, but also on cellular adhesion and migration in the context of the CLL microenvironment [12, 27, 28, 54]. We indeed observed that these inhibitors reduced EMC cell line proliferation and viability, as well as adhesion towards fibronectin. The reduced survival of EMC cell lines was due to inhibition of PI3K/Akt/Survivin signaling, as also shown for human CLL [50]. Although we did not find evidence for a role of Bcl-2 family members in the outgrowth of the EMC cell lines, upon treatment with the Bcl-2 inhibitor ABT-199 the EMC cell lines also underwent apoptosis (S.P.S. unpublished results). Thus, next to exhibiting a similar phenotype to human CLL, the EMC cell lines also recapitulate key responses to kinase inhibition therapies *in vitro*. We conclude that the EMC cell lines are well suited for high-throughput screening for efficacy or studies on the mechanism of action of novel compounds combinations for CLL treatment. Moreover, due to the transferability of EMC cells into *Rag1^−/−^* mice *in vivo* responses can be evaluated as well. The rapid CLL development in these engrafted mice facilitates the evaluation of therapeutic strategies within reasonable time schedules and contrasts with the slow disease development in CLL mouse models [33, 35, 37]. Unfortunately, we have been unsuccessful in establishing EMC cell engraftment in WT mice, which might be related to their mixed C57Bl6×129 background. Nevertheless, ibrutinib treatment of EMC-engrafted *Rag1^−/−^* mice resulted in increased circulating CLL cell numbers and concurrently decreased tumor load in the spleen, as well as prolonged survival, thus recapitulating key features of ibrutinib therapy in clinical studies of relapsed/refractory CLL [12].

Patients harboring del(17p13.1) or *TP53* mutations represent a difficult to treat CLL subgroup, warranting development of novel targeted agents. Our EMC cell lines likely reflect a CLL subtype with dysfunctional p53 due to interaction with SV40 large T protein [37, 55, 56]. This was further supported by a lack of induction of p53 target gene PUMA upon fludarabine treatment. Therefore, EMC cell lines specifically provide a tool to dissect novel treatment options for CLL with a dysfunctional p53 pathway.

In summary, we have generated stable monoclonal cell lines from a CLL mouse model that exhibits constitutive Btk and Akt signaling, presents several features of human CLL and responds to novel targeted therapies. These EMC cell lines thus provide a novel *in vitro* and *in vivo* preclinical platform to study CLL cell biology and to test efficacy of novel targeted therapy combinations.

## MATERIALS AND METHODS

### EMC cell line culture

Single-cell suspensions (1 × 10^6^ cells/ml) obtained from spleen from *IgH.TE*μ mice, which were on a mixed C57Bl/6 × sv129 background (EMC6) or on the *Aicd^−/−^* (C57Bl/6 × sv129) background [57] (EMC2 and EMC4). These *IgH.TE*μ mice were diagnosed with leukemia on the basis of a high tumor load (> 90% CD5^+^CD43^+^IgM^+^CD19^+^ cells) in peripheral blood. Leukemic cells were cultured in medium (RPMI 1640, 10% FCS, 50 ug/ml gentamycin, 50 μM 2-mercapto-ethanol, all components from Life Technologies^TM^), under various conditions, with or without BAFF (25ng/ml, R&D Systems), α-CD40 antibodies (20 ug/ml, R&D Systems), rIL-4 (50 ng/ml, Peprotech) and incubated at 37°C in the presence of 5% CO_2_. After initial passages, the EMC cell lines continued to expand in the absence of added growth factors and therefore the cell lines were propagated in culture medium only. For optimum growth, the EMC4 and EMC6 cell lines were propagated in dilution of 0.25 × 10^6^ cells/ml and EMC2 cell lines were propagated in dilution of 0.5 × 10^6^ cells/ml. Once growing in culture the doubling time for the EMC6 and EMC4 lines were ∼36 hours and for EMC2 ∼72 hours. The culture media were refreshed twice a week. The cultures were stopped after 12 weeks of the initial culture and vials were frozen. For subsequent experiments, vials were thawed and expanded. All experiments were performed one day after refreshing medium and expanding cell culture. For all *in vitro* assays cells were plated in dilution of 1×10^6^ cells/ml.

### Adoptive transfer into Rag1^−/−^ mice and ibrutinib treatment

For adoptive transfer, 5 × 10^6^ EMC4 cells or 1 × 10^6^ EMC6 cells were injected intraperitoneally (i.p.) into *Rag1^−/−^* mice [51]. Mice were monitored for leukemia development by regular blood screening for the presence of CD5^+^CD43^+^IgM^+^CD19^+^ leukemic cells: at day 7, 10 and 14 after engraftment subgroups of mice were tested. Mice were euthanized when they developed signs of sickness, such as lethargy, aversion to activity, shallow or labored breathing and other disabling symptoms.

Ibrutinib (Chiralstar, USA) treatment was initiated 2 weeks following engraftment in mice with > 2% leukemic cells in the circulation, as detected by flow cytometry. Mice were randomized into ibrutinib and vehicle treatment group and ibrutinib (25 mg/kg in water/5%mannitol/0.5%gelatin) or vehicle was orally administered to the mice once daily for 16 days. Mice were euthanized when they developed signs of severe disease, as described above, or at the end of the 16-day treatment cycle.

Mice were housed at the Erasmus MC experimental animal care facility under specific pathogen-free conditions. Animal procedures were reviewed and approved by the Erasmus MC Animal Experiments Committee.

### Flow cytometry procedures

Preparation of single-cell suspensions of lymphoid organs and lysis of red blood cells were performed according to standard procedures. Cells were directly stained in the appropriate buffer using the following fluorochrome-conjugated monoclonal antibodies: anti-CD19 (1D3, eBioscience), anti-CD5 (53-7.3, eBioscience), anti-CD43 (S7, BD), anti-IgD (11-26, BD), anti-IgM (Il/41, eBioscience), anti-CD3 (17A2, eBioscience), anti-CD11b (M1/70, eBioscience), anti-MHCII (M5/114.15.3, eBioscience), anti-CD86 (GL1, BD), anti-CD69 (H1.2F3, eBioscience), anti-CD25 (PC61.5, eBioscience), anti-CXCR4 (2B11, BD), anti-CXCR5 (2G8, BD), anti-CCR7 (4B12, eBioscience). All flow cytometric measurements were performed on a LSRII flow cytometer (BD Biosciences) and results were analyzed using FlowJo-V10 software (TreeStar).

For intracellular flow cytometry analysis of phosphorylated proteins (Phosflow), cells were starved for 10 min at 37°C in FCS-free “RPMI-plus” medium (RPMI 1640 from Life Technologies^TM^) and subsequently stimulated with 20 μg/ml goat anti-mouse F(ab’)_2_ anti-IgM fragments (Jackson Immunoresearch) for 5 min (for p-Btk, p-Slp65, p-Plcγ2) or 3 hrs (for p-Akt, p-S6). Unstimulated control cells were treated in parallel without F(ab’)_2_ α-IgM. Following stimulation, cells were fixed in Cytofix fixation buffer (BD Bioscience) for 10 min at 37°C and permeabilized with Perm Buffer III (BD Biosciences) at −20°C for 30 min. The cells were then stained with either with fluorochrome-conjugated anti-p-Btk(Y551), anti-p-Btk(Y223), anti-p-Slp65/BLNK(Y84), anti-p-Plc-y2 (Y759) antibodies (all from BD Biosciences) or with unconjugated anti-p-Akt(S473), anti-p-S6 (all from Cell Signaling Technology) and PE-conjugated secondary antibody (Jackson ImmunoResearch). WT splenic cell suspensions were stained extracellularly with anti-B220 (RA36B2, eBioscience) and CD3 (145-2c11, BD) before fixation.

### Calcium (Ca^2+^) flux assays

Intracellular Ca^2+^ flux was measured using the Fluo3-AM and Fura Red-AM fluorogenic probes (Life Technologies), as previously detailed [19]. In brief, mouse splenocytes (5 × 10^6^) were incubated with 5 μM Fluo3-AM and 5 μM Fura Red-AM in loading buffer (HBSS medium supplemented with 10 mM HEPES and 5%FCS) at 37°C for 30 min in the dark. To gate for untouched B cells in WT splenocytes, we added biotinylated Abs to NK1.1 (PK136, BD), CD4 (GK1.5, BD), CD8a (53–6.7, BD), Ter119 (BD), CD11c (N418, ebiosciences), Gr-1 (RB6-8C5, ebiosciences), and FcRεI (MAR-1, ebiosciences) for the final 10 min of incubation. Cells were subsequently washed and stained with fluorochrome-conjugated streptavidin at RT for 10 min as a second step for biotin-conjugated antibodies. Cells were then washed, resuspended in buffer (HBSS medium with 10 mM HEPES, 5%FCS and 1 mM CaCl_2_), filtered and left for at least 30 min at RT. Cells were warmed to 37°C for 5 min before acquisition of events.

Basal intracellular Ca^2+^ levels were measured for 60 s, followed by BCR stimulation with either 20 μg/ml goat anti-mouse F(ab’)_2_ anti-IgM fragments or plain control liposomes (DOPC/CHOL 55:45, Formumax Scientific Inc.) and measured for 5–7 min. At the end of each Ca^2+^ measurement, cells were stimulated with 4 μg/ml ionomycin (Life Technologies, Carlsbad, California, USA) to measure maximum Ca^2+^ signaling.

To determine effects of ibrutinib on Ca^2+^ mobilization, 5 × 10^6^ EMC cells and WT splenocytes were pre-incubated in culture medium with or without ibrutinib (1 μM) at 37°C for 3 h. Staining and Ca^2+^ mobilization measurements were performed as described above.

### Cell cycle and viability assays

To evaluate the effect of ibrutinib on survival and proliferation, 1 × 10^6^ EMC cells were placed in culture medium, with or without the appropriate concentration of ibrutinib at 37°C for 24 h. Following treatment, cells were fixed with ethanol and stained with propidium iodide, according to the instructions of the manufacturer (Life Technologies^TM^). Cell cycle analysis was performed as previously described [58].

For testing sensitivity to chemotherapeutic agents, EMC cell lines were incubated with different concentrations of Etoposide, Cisplatin or Fludarabine from Sigma Chemical (St.Louis, MO) for 24 h. Viability was measured by DiOC6/PI staining as described [59]. Relative viability was defined as [% viable cells treated condition/ % viable cells in medium control] × 100.

### Adhesion assay

EMC Cells were treated with either ibrutinib (10 nM) or idelalisib (100 nM) or a combination and allowed to adhere to fibronectin-coated surfaces and adhesion to fibronectin-coated plates was measured as described previously [27].

### RNA sequencing from naïve, activated and CLL B cells

Splenic single-cell suspensions were prepared in magnetic-activated cell sorting (MACS) buffer (PBS/2 mM EDTA/0.5%BSA) and naïve splenic B cells from 8–12 week-old WT C57BL/6 mice were purified by MACS, as previously described [60]. Non-B cells, B-1 cells, germinal center B cells and plasma cells were first labeled with biotinylated antibodies (BD Biosciences) to CD5 (53–7.3), CD11b (M1-70), CD43 (S7), CD95 (Jo2), CD138 (281-2), Gr-1 (RB6-8C5) and TER-119 (PK136) and subsequently with streptavidin-conjugated magnetic beads (Miltenyi Biotec). Purity of MACS-sorted naïve B cells was confirmed by flow cytometry (typically > 99% CD19^+^ cells). To obtain activated B cells, purified naïve B cells were cultured in RPMI culture medium in the presence of 10 μg/ml F(ab’)_2_ anti-IgM (Jackson Immunoresearch) for 12 h.

RNA was extracted from naive or activated splenic B cells, as well as from purified EMC2, EMC4 and EMC6 primary tumors (using MACS-purification for CD19^+^ cells) and from the three EMC cell lines from *IgH.TEμ* mice with Qiagen's RNeasy Mini and Micro kits according to manufacturer's instructions followed by DNase treatment. To facilitate comparisons with resting and activated B cells, MACS purified naïve mature splenic B cells from WT mice, either directly or after 12 hours of stimulation with a-IgM were included. The purity of sorted cells (typically > 99% CD19^+^ cells) was confirmed by flow cytometry.

Total mRNA sequencing was performed on a HiSeq 2500 (Illumina), and raw reads were aligned using Bowtie to murine transcripts (RefSeq) corresponding to the University of California at Santa Cruz (UCSC) mouse genome annotation (NCBI37/mm9) [61]. Statistical analysis of the RNAseq data was performed using HTseq and edgeR. Gene counts were computed using HTseqCount [62]. Differential gene expression analysis was performed using EdgeR with a false discovery rate (FDR) < 0.05 and a log2-fold change cutoff of 1 [61]. Gene counts were converted to logCPM (log counts per million) values for principle component analysis (PCA), which was performed in R, a language for statistical computing (http://www.r-project.org). For generating heatmaps and molecular pathway enrichments the differentially expressed gene list was further filtered for genes with an absolute FPKM value of >1 in at least 2 samples in either group. Molecular pathway enrichments were obtained from the MSigDB [45].

### Quantitative real time (RT-) PCR analysis

For quantitative RT-PCR analysis, TaqMan probes were employed. Probe Finder software (Roche Applied Science), the Universal Probe Library (Roche Applied Science) and Ensembl genome browser (http://www.ensembl.org/) were used for primer and probe design. Taqman Universal Master Mix II, was purchased from Thermo Fisher Scientific. For 15 μl RT-PCR reaction, 7.5 μl master mix, 4.15 μl nuclease-free water, 0.6 μl forward and reverse primer (10pmol/ul) and 0.15μl probe was added to the cDNA per reaction. Quantitative RT-PCR was performed by using the 7300 Real Time PCR system (Applied Biosciences) according to manufacturer's instructions. Gene expression was analyzed with an ABI Prism 7300 Sequence Detector and ABI Prism Sequence Detection Software version 1.4 (Applied Biosystems). Cycle-threshold levels were calculated for each gene and housekeeping gene glyceraldehyde-3-phosphate dehydrogenase (Gapdh) was used for normalization of the values. All primer sequences and probe numbers are listed in Supplementary Table 2.

### Statistical analysis

Statistical analysis was performed using GraphPad Prism software (San Diego, California, USA). For calculating levels of significance, for differences between groups of continuous data we used Mann-Whitney *U* test. To compute synergistic effects of combination treatments we used CompuSyn (version 1.0) software based on Chou and Talalay method [49]. The log rank test was used for calculating the level of significance for survival differences between differently treated mice groups. The one-sample *T*-test was used to determine the significance of differences between means and normalized values (100%).

## SUPPLEMENTARY MATERIALS FIGURES AND TABLES


