# Supplementary material for: Cell lines generated from a chronic lymphocytic leukemia mouse model exhibit constitutive Btk and Akt signaling
Source: Oncotarget. 2017 May 26;8(42):71981–95. doi: 10.18632/oncotarget.18234 (PMC5641105; doi:10.18632/oncotarget.18234)
Supplement: Supplementary file 1 [file oncotarget-08-71981-s001.pdf]

Cell lines generated from a chronic lymphocytic leukemia mouse model exhibit constitutive Btk and Akt signaling

Supplementary Materials

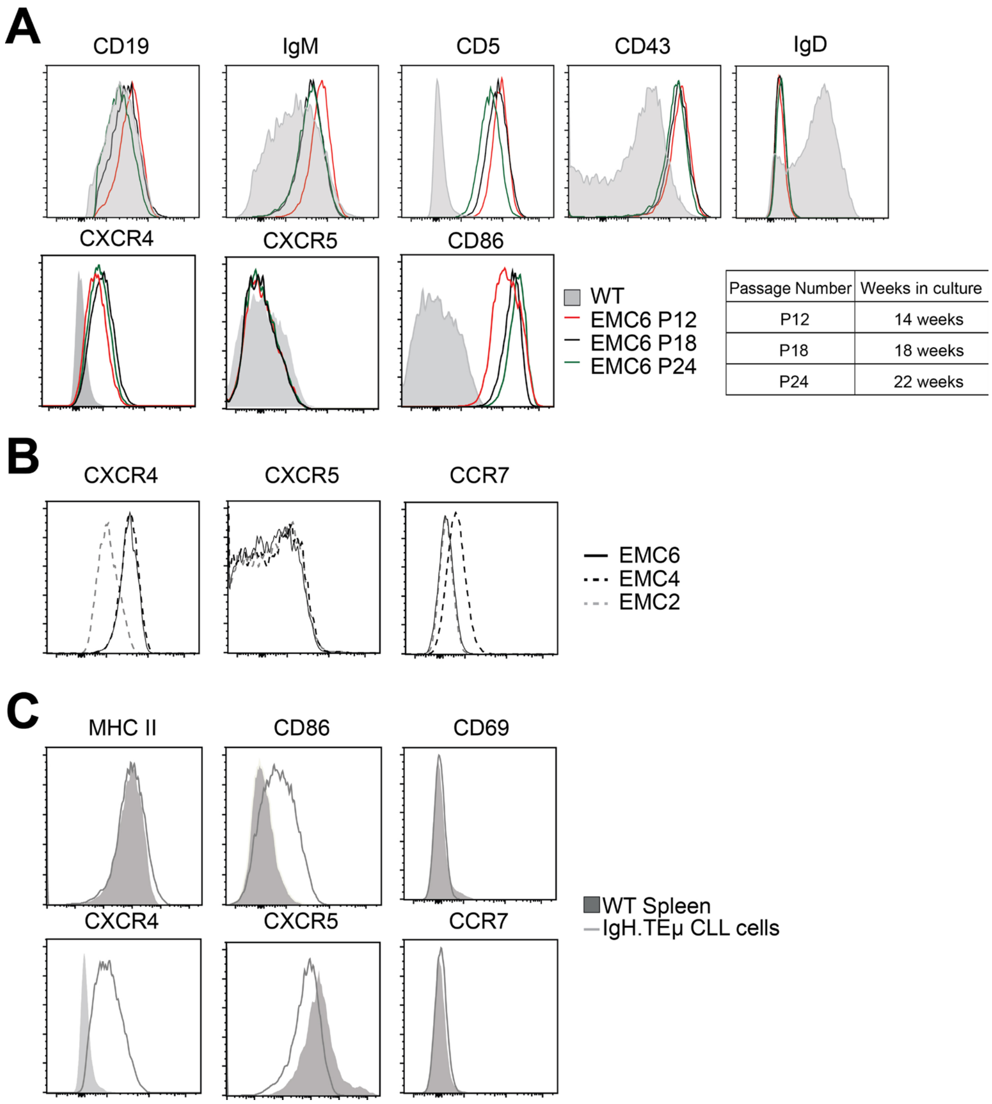

Supplementary Figure 1: EMC cell lines resemble primary tumors from IgH.TEμ mice. (**A**) Histograms showing surface expression of the phenotypic markers on EMC6 cells, as determined by flow cytometry, over the indicated culture period. EMC4 and EMC2 showed similar stable expression profiles. (**B**) Comparison of expression of indicated chemokine receptors on three different EMC cell lines by flow cytometry. (**C**) Representative plot for expression of indicated activation markers and chemokine receptors on gated CD19^{+} WT splenic B cells and gated CD5^{+}CD19^{+}CD11b^{-}CD43^{+} CLL B cells from an aged *IgH.TEμ* mouse (representing n > 20) as determined by flow cytometry.

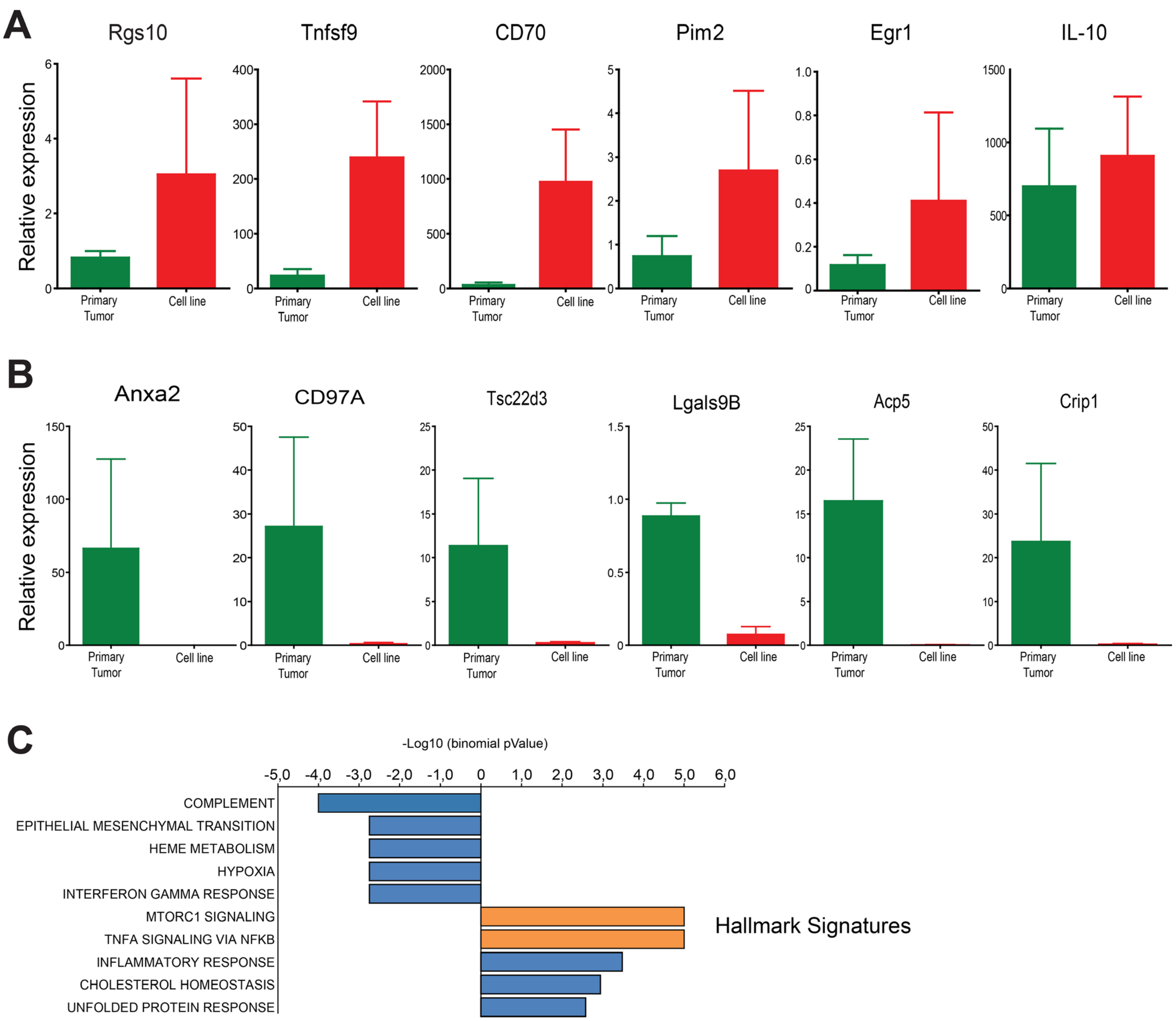

D

Genes	Primary Tumors (n=3)	EMC cell lines (n=3)	Control WT B cells	α -IgM Control WT B cells
Bcl-XL	5.33 ± 2.27	11.03 ± 5.72	2.30 ± 0.30	44.3 ± 1.10
Bcl-2	20.6 ± 3.77	15.37 ± 2.93	19.3 ± 1.68	7.52 ± 0.35
Mcl-1	258.9 ± 2.56	236.5 ± 21.90	130.8 ± 9.77	123.1 ± 3.68
Survivin	79.03 ± 20.05	102.8 ± 14.69	2.90 ± 0.43	3.02 ± 0.34

Supplementary Figure 2: RNA-sequencing reveals active pathways in EMC cell lines. (**A**, **B**) Validation of RNA-sequencing data by real time quantitative PCR. Bars represents mean values ± SEM expression of indicated genes, either upregulated (A) or downregulated (B) in cell lines (n = 3) compared to original primary tumor (n = 3) from *IgH.TEμ* mice. The expression values were calculated relative to expression in splenic B cells from wild type mice (n = 4), which were set to 1. (**C**) Hallmark Signature analysis of pathway enrichment for genes that show differential expression in EMC cell lines compared to original primary tumors from *IgH.TEμ* mice. (**D**) Expression values (FPKMs) for indicated anti-apoptotic genes in original primary tumor (n = 3) from *IgH.TEμ* mice, EMC cell lines (n = 3), unstimulated (n = 4) and F(ab')2 anti-IgM stimulated (n = 4) control WT splenic B cells compared to corresponding primary tumor.

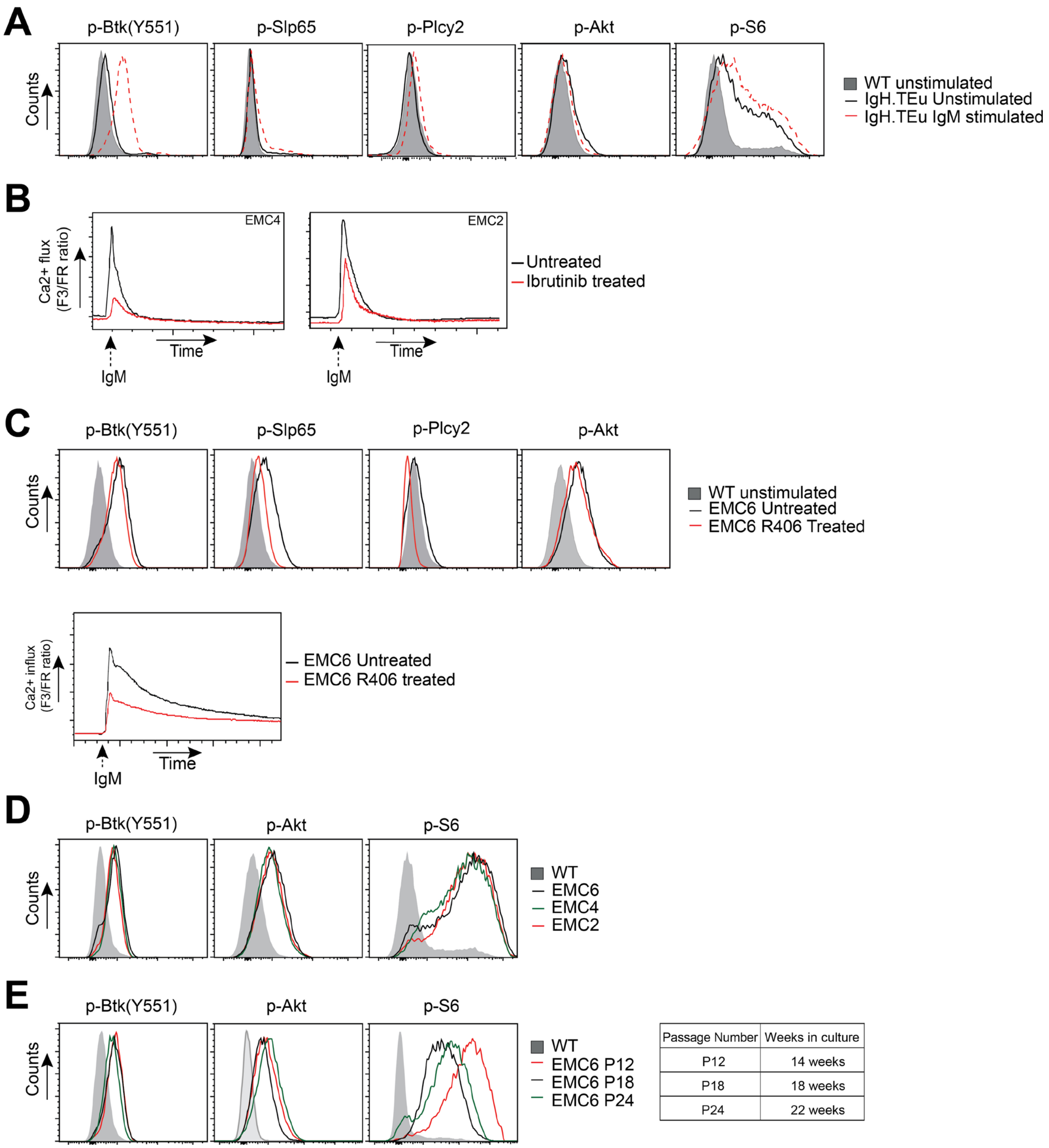

Supplementary Figure 3: EMC cell lines and IgH.TEm-derived primary tumor cells exhibit constitutive active BCR signaling. (**A**) Comparison of Ca^{2+} influx between untreated cells (black) and following 3 hours in-vitro treatment with 1mM Ibrutinib of the indicated EMC cell line. The dotted arrow indicate addition of anti-IgM. (**B**) Flow cytometry analysis of the indicated phosphoproteins on gated $B220^{+}CD3^{-}$ wild type (WT) splenocytes (shaded area), unstimulated (solid black line) and F(ab')2 anti-IgM stimulated (20 ug/ml, dotted red line) CLL cells from aged *IgH.TEμ* mice. (**C**) *Top*: Flow cytometry analysis of the indicated phosphoproteins on gated $B220^{+}CD3^{-}$ WT splenocytes (shaded area), untreated EMC6 (solid black line) and R406-treated (4 mM of Syk inhibitor R406, solid red line) EMC6 cells. *Bottom*: Comparison of Ca^{2+} influx between untreated EMC6 cells or EMC6 following *in vitro* treatment with 4 mM Syk inhibitor (R406). The dotted arrow indicates addition of F(ab')2 anti-IgM. (**D**) PhosFlow analysis of the indicated phospho-proteins expressed in the three EMC cell lines, as well as gated unstimulated $B220^{+}CD3^{-}$ WT splenocytes, as a reference. The histogram plots shown are representative of three independent experiments. (**E**) PhosFlow analysis of the indicated phospho-proteins expressed in the EMC6 cell line, showing its stability over the indicated culture periods. Gated unstimulated $B220^{+}CD3^{-}$ WT splenocytes serve as a reference.

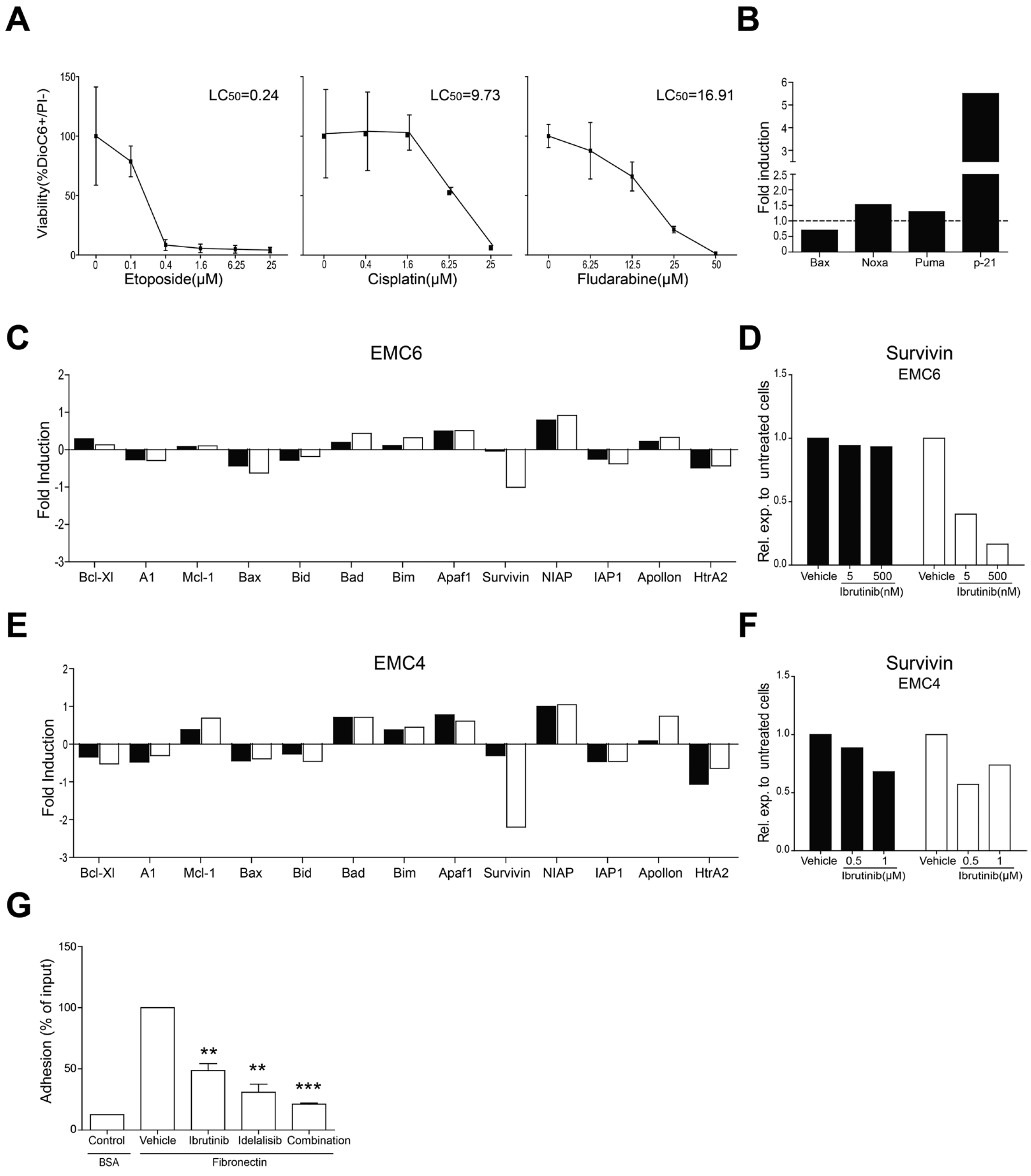

Supplementary Figure 4: CLL cell lines are sensitive to chemotherapeutic drugs. (**A**) EMC4 cells were cultured in the presence of the indicated concentrations of Etoposide, Cisplatin or Fludarabine for 24 hrs. The sensitivity (LC50) towards individual agent is shown. (**B**) Bar graphs represent Multiplex Ligation-dependent Probe Amplification (MLPA) analysis for respective genes in EMC4 cell line following treatment with Fludarabine for 24 hours. (**C**, **E**) Fold induction (^{2}log) of mRNA levels of indicated pro-/anti-apoptotic mediators in the indicated cell lines upon *in vitro* treatment with Ibrutinib (EMC6 = 5 nM; EMC4 = 500 uM) for 12 hrs (*filled black bars*) or 24 hrs (*clear white bars*). Fold induction was calculated with respect to vehicle treated cell lines. (**D**, **F**) Validation of MLPA result by real time quantitative PCR on survivin upon *in vitro* treatment of the indicated cell line with ibrutinib (dose indicated) for 12 hrs (*filled black bars*) or 24 hrs (*clear white bars*). (**G**) *In vitro* adhesion assay: EMC4 cells pretreated with either ibrutinib (10 nM) or idelalisib (100 nM) or a combination were allowed to adhere to fibronectin-coated surfaces (n = 3, in triplicate). Graphs are presented as normalized mean ± SD (100%= EMC4 cells treated with vehicle). $^{**}P < 0.01$ $^{***}P < 0.0001$ (paired one-sample T-test) .

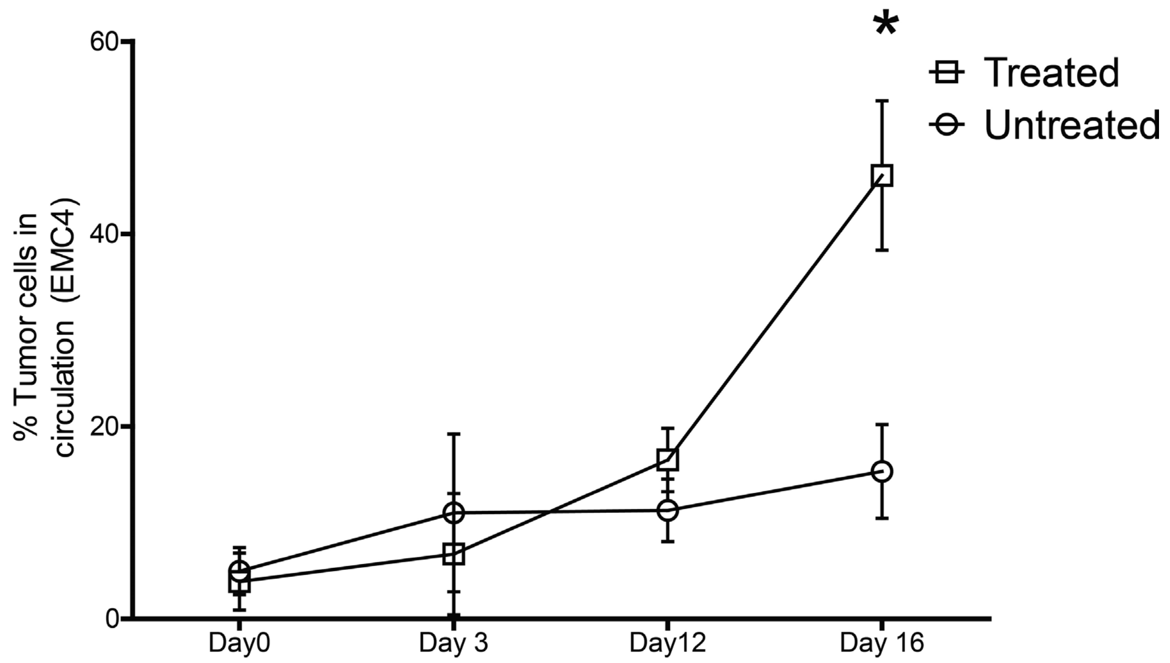

Supplementary Figure 5: Rapid induction of CLL following engraftment of CLL cell line. Proportions of $CD5^+CD19^+CD43^+$ tumor cells in peripheral blood from EMC4 engrafted *Rag1*$^{-/-}$ mice (*n* = 4 per group) following treatment with either vehicle (open circles) or ibrutinib (open squares) for the indicated time. The treatment was started 14 days following engraftment. Result show mean ± SEM.

Supplementary Table 1: (A) Expression values (FPKMs) for various genes upregulated in EMC cell lines compared to corresponding primary tumor

Genes	EMC6 PT	EMC2 PT	EMC4 PT	EMC6 line	EMC2 line	EMC4 line	*p* Value
Insig1	12,2	9,0	6,9	52,7	38,0	39,5	0,00019069
Pim2	22,1	39,8	43,9	122,9	143,7	229,1	0,0001479
Fam179a	0,3	0,3	5,3	5,1	3,6	20,2	0,00041553
Rgs10	26,2	8,4	9,7	151,5	74,6	26,5	0,00060558
Sc4mol	8,8	8,7	6,6	55,3	29,6	65,1	2,0556E-05
Sqle	8,4	4,4	8,0	46,5	25,7	60,0	1,7405E-05
Dusp4	0,3	0,7	2,8	6,6	4,4	13,1	0,00043918
Il10	10,2	31,5	88,2	155,3	125,4	551,9	2,4666E-06
Col11a2	0,7	1,5	1,4	6,3	8,7	8,8	3,4153E-05
Egr1	53,9	23,0	30,3	179,3	394,6	159,7	1,6531E-05
Ncan	0,1	0,1	1,0	5,1	1,9	3,2	0,00055946
Wnt10a	3,0	0,5	0,9	29,6	18,9	8,7	7,5123E-07
2310033E01Rik	0,0	0,6	2,8	4,0	25,6	17,3	0,00018339
Ccdc88a	0,1	0,2	0,4	5,9	2,2	2,3	1,073E-05
Asns	0,1	2,5	16,8	3,6	1,4	287,3	0,00012651
Slc2a6	1,0	4,1	3,5	58,8	49,1	27,4	2,2787E-08
Spna1	0,0	0,1	1,5	1,1	0,3	24,4	3,9303E-06
Axin2	0,0	0,0	1,5	1,8	19,8	4,1	3,8398E-07
Dmrta2	0,2	0,1	0,6	5,3	3,4	6,7	2,3009E-05
Lag3	1,2	0,5	2,2	5,8	6,8	54,4	6,0572E-05
Hapln4	0,0	0,1	0,3	2,8	2,6	2,8	3,8145E-05
Sytl1	0,4	1,4	2,0	22,4	8,2	57,2	7,5952E-09
Gpr162	0,0	0,2	0,5	0,6	13,1	2,4	4,7813E-05
Rab39b	0,0	0,1	0,1	1,0	0,2	3,7	0,00035858
Gpr171	0,0	0,1	0,4	0,9	0,3	12,0	0,00039336
Tnfsf9	5,4	1,8	1,5	100,4	84,1	66,5	2,1113E-17
Fads2	0,0	0,1	0,2	6,1	1,5	6,7	3,9377E-05
Mpp2	0,0	0,0	0,1	2,2	1,2	0,2	0,00070589
Dapk2	0,1	0,7	0,3	14,4	21,2	8,7	2,2963E-08
Hnrpll	0,2	0,2	0,3	22,6	0,2	3,8	0,00011817
Csgalnact1	0,0	0,0	0,1	4,2	0,0	2,5	0,00054088
Chac1	0,0	0,5	0,3	2,8	0,5	36,8	0,00011808
Arhgef25	0,0	0,0	0,1	1,7	0,5	4,1	6,7117E-05
Cd70	1,2	0,0	1,3	63,9	39,5	47,7	1,7725E-08

Supplementary Table 1: (B) Expression values (FPKMs) for various genes downregulated in EMC cell lines compared to corresponding primary tumor

Genes	EMC6 PT	EMC2 PT	EMC4 PT	EMC6 line	EMC2 line	EMC4 line	*P* value
Gda	0,1	1,2	1,7	0,0	0,0	0,0	7,1799E-08
Iigp1	1,9	0,4	1,5	0,0	0,0	0,0	1,3359E-07
Pla2g7	0,1	1,8	2,4	0,0	0,0	0,0	2,9417E-06
Lrg1	0,8	2,1	1,9	0,0	0,0	0,0	5,015E-07
Clec4n	0,3	2,5	3,9	0,0	0,0	0,0	1,2037E-06
Gtsf1	3,4	2,3	4,4	0,0	0,0	0,0	4,6376E-07
C1qc	0,7	2,3	17,4	0,0	0,0	0,0	4,0055E-08
C1qb	1,0	2,8	22,3	0,0	0,0	0,0	4,9175E-09
S100a9	0,0	1,5	43,0	0,0	0,0	0,0	2,7688E-05
S100a8	0,9	2,1	60,4	0,0	0,0	0,0	9,695E-06
Lyz2	1,9	95,4	34,9	0,0	0,0	0,0	2,3255E-12
S100a6	28,9	724,6	48,2	0,0	0,6	0,1	2,0847E-11
C1qa	0,7	4,2	18,7	0,0	0,0	0,0	7,019E-07
Lgr5	6,6	0,4	1,2	0,0	0,0	0,0	1,1054E-09
Tgfbi	1,3	1,5	4,3	0,0	0,0	0,0	1,4671E-08
Gpx7	7,8	0,2	6,2	0,0	0,0	0,0	4,1037E-06
S100a4	4,2	40,7	14,0	0,0	0,1	0,0	4,8877E-07
Vcam1	0,3	2,2	5,7	0,0	0,0	0,0	7,6093E-07
AI427809	1,9	1,8	0,3	0,0	0,0	0,0	2,0367E-06
2610018G03Rik	3,3	12,1	2,9	0,0	0,1	0,0	8,3176E-09
Timd4	0,0	1,5	1,6	0,0	0,0	0,0	0,000145
Anxa2	12,2	224,3	14,4	1,0	0,1	0,1	3,2995E-11
Slc4a1	0,1	1,9	4,4	0,0	0,0	0,0	1,0717E-06
Plbd1	0,5	7,1	3,0	0,0	0,0	0,0	1,143E-06
Npr3	0,9	1,5	1,1	0,0	0,0	0,0	4,2053E-07
Art3	5,9	0,2	1,1	0,1	0,0	0,0	0,00013629
Pag1	1,4	11,1	0,2	0,0	0,1	0,1	8,929E-07
Cd5l	0,8	3,3	8,3	0,0	0,1	0,0	5,5605E-06
Gm11428	1,6	1,0	51,0	0,0	0,0	0,7	0,00013158
Hebp1	0,3	2,3	7,0	0,0	0,0	0,1	4,4198E-05
Smpdl3a	4,8	1,8	6,9	0,1	0,0	0,1	9,8492E-06
Tppp	2,0	15,3	1,2	0,1	0,1	0,0	3,738E-08
Tnfsf4	0,3	11,6	2,3	0,0	0,2	0,0	0,00019424
Csf1r	0,3	4,4	4,0	0,0	0,0	0,1	7,8117E-07
Ifitm3	1,4	3,4	17,8	0,3	0,1	0,2	0,00021669
Pcp4	57,4	18,2	6,4	0,5	0,2	1,7	0,00068174
Tppp3	3,7	75,4	4,5	0,7	0,8	0,9	2,9088E-05
Galnt10	3,6	2,3	3,6	0,2	0,0	0,1	9,9806E-06
Cacna1h	22,2	3,7	9,1	0,2	0,0	1,1	6,3115E-05
Rnf125	4,3	1,8	1,4	0,2	0,0	0,1	0,0006327
March1	12,5	36,5	10,8	0,1	2,4	0,1	9,8741E-05
Ildr1	0,2	4,6	1,3	0,0	0,0	0,3	0,00018409
Dnahc8	1,7	2,0	5,1	0,1	0,3	0,0	5,6348E-05
Tsc22d3	61,7	304,7	82,3	7,2	11,4	8,2	5,1506E-08
Dkkl1	5,4	38,1	3,3	2,2	0,5	0,3	0,0002477
B3gnt5	17,4	44,0	13,3	0,2	4,4	0,4	0,00022842
Ggh	15,9	6,6	0,4	1,6	0,0	0,0	0,00015714
Abca1	1,8	12,2	4,3	1,1	0,1	0,2	4,5105E-05
Cacna1d	6,1	2,2	1,2	0,6	0,0	0,1	0,00013495
Acp5	44,4	85,3	82,2	2,6	9,4	4,7	9,6697E-08
Emb	0,7	51,8	13,6	5,2	0,3	0,1	0,00030886
Crip1	1479,0	2936,6	396,2	686,8	8,4	57,6	0,00037951
Cd97	129,1	387,3	75,1	31,8	23,4	38,3	0,00017688
Sepp1	0,7	7,0	66,6	0,0	0,8	11,5	1,3934E-05
Nedd4	46,1	17,6	33,3	4,4	10,1	2,1	0,0006947
Zfp36l2	36,4	100,6	68,0	14,4	12,5	9,2	0,00011901
Lgals9	30,6	28,5	31,2	3,8	2,9	9,6	0,00056366
Arhgef3	13,7	26,7	13,1	0,9	1,9	7,5	0,00026268
Mylip	20,2	22,5	6,3	7,3	1,6	0,7	0,0003681
Gm4759	7,1	4,0	3,2	1,3	1,4	0,5	0,00021789
Cntln	0,0	3,2	6,8	0,0	0,0	2,5	0,00056445
Plk2	0,1	10,1	14,1	0,0	0,1	6,7	0,00039975

Supplementary Table 2: List of primers and probes used in real-time quantitative PCR

Gene	Forward primer sequence (5′ to 3′)	Reverse primer sequence (5′ to 3′)	Universal Probe library probe:
Tnfsf9	cgccaagctactggctaaaa	cgtacctcagaccttgagataggt	#33 (cat. no. 04687663001)
Pim2	tcagcgggctcaatatacg	gaaagctgcccgatcctt	#25 (cat. no. 04686993001)
Rgs10	cacaccctctgatgttccaa	gaagcggctgtagctgtcat	#1 (cat. no. 04684974001)
CD70	gtccttcacacacggacca	aggccatcttgatggatacg	#25 (cat. no. 04686993001)
Egr1	cctatgagcacctgaccaca	tcgtttggctgggataactc	#22 (cat. no. 04686969001)
IL-10	actgcacccacttcccagt	tgtccagctggtcctttgtt	#21 (cat. no. 04686942001)
Anxa2	tgtccacgaaatcctgtgc	taggcacttgggggtgtaga	#64 (cat. no. 04688635001)
CD97	ctgcagggcctattcctcta	gcaggcccatttccagta	#13 (cat. no. 04685121001)
Acp5	cgtctctgcacagattgcat	aagcgcaaacggtagtaagg	#60 (cat. no. 04688589001)
Crip1	gctgagagccacactttcaa	ttaaaggcactgagggttcc	#56 (cat. no. 04688538001)
Tsc22d3	ggtggccctagacaacaaga	tcaagcagctcacgaatctg	#10 (cat. no. 04685091001)
Lgals9	ccaggggactaccaagagttt	cttcgtgttgcaaaccacat	#12 (cat. no. 04685113001)

Supplementary Table 3: Basal Calcium flux in the three EMC cell lines

	Basal Ca^{2+} Flux (F3/FR ratio, mean ± SEM)	Mann-Whitney *U* test *P* Value
WT Splenic B cells	0.09 ± 0.01	-
EMC6	0.38 ± 0.17	0.0095
EMC4	0.19 ± 0.06	0.0082
EMC2	0.15 ± 0.01	0.0571

The significance value in the three EMC cell lines is calculated w.r.t. WT splenic B cells in three independent experiments.
